# Poisoning by Arsenic

**Published:** 1846-11

**Authors:** 


*Poisoning by Arsenic.*—The widow M——, and Antoine B—— were recently tried at the assizes of Herault for the double crime of adultery and poisoning the husband of the female prisoner. The facts, as they appeared in evidence, were as follows:—In August, 1844, shortly after several violent quarrels between M—— and his wife, caused by the intimacy between the latter and B——, and just at the period when B——, because of the consequent scandal, had lost his only means of subsistence, M—— fell sick. The symptoms of his malady were shiverings, cold perspirations, great itching in the extremities, and at length considerable emaciation. As no suspicion was then excited, and as the symptoms presented periods of subsidence which simulated intermission, they were considered as the effect of accesses of fever, especially as intermittent fever is endemic in the locality, and his wife was actually labouring under that disease. The wife, becoming convalescent, paid a visit to her family, and during her absence, her husband, whose malady had been so serious that he sought for the consolations of the church, became convalescent also. On the return of the wife the husband's malady again became aggravated, and continued until the vintage, at which period the wife again left home to superintend some matters of business, and during her absence, which continued a fortnight, the husband's health was so far re-established that he was able to look after his affairs. His physicians then considered that he had only to continue the prescribed regimen to become perfectly restored to health. From this period M——— continued in the country with his wife, but his convalescence, instead of being progressive, was suddenly interrupted. For a few days he felt tolerably well, but then his former sufferings commenced to return. New symptoms were also gradually superadded; vomiting occurred several times, and the wife on each occasion threw the ejected matter out of the window. On one occasion M——— vomited on the floor, and the wife scattered ashes on what was thrown from the stomach, and subsequently washed the floor with potash.

Nothing as yet, however, indicated that M——— was near his death. Early in December he attended to his affairs as usual; but on the 5th of that month, after a short walk, he became chilly, and took to his bed in the afternoon. From that moment his wife never left him, and no one else entered his chamber.

On the 6th December M——— died, no one being present, says the wife, who informed the inmates of the house that her husband had just expired tranquilly, and without convulsions. The inferior extremities, however, were found contracted, the body was already cold and cadaveric, rigidity so great that it was impossible to remove the clothes completely.

Some vague suspicions at first existed, but soon subsided, until they subsequently became revived and strengthened from a train of circumstances unnecessary to be detailed. The authorities then directed the body to be exhumed, when the usual fact was observed, that the extremities were completely decomposed, while the parietes and viscera of the abdomen, which usually putrefy first, were in a state of perfect preservation. This circumstance, together with the existence of some yellow stains, such as might be produced by the action of certain products of putrefaction on a compound of arsenic, raised a suspicion that M——— might have been poisoned by such a substance. This suspicion was converted into a certainty by chemical analysis. Arsenic was detected in the intestines, in the liver, in the spleen, in the kidneys, in the bladder, in the heart, in the lungs, in the muscles of the stomach and of the abdomen, as also in the liquid contents of the hollow abdominal viscera.

As M——— had vomited several times, and his wife had disposed of the material vomited by throwing it from a window, and washing the floor, arsenic was sought for in those situations. The soil in the immediate vicinity of the window and also at a certain distance from it, was collected and analysed, as were scrapings from various parts of the floor of the chamber. Arsenic was detected to a marked amount in the soil collected next the wall immediately under the window, and also, but in a much smaller quantity, in that collected within the space of 8 or 10 feet from that point. All the scrapings from those parts of the floor which were stained with the material rejected by vomiting yielded arsenic, but not a trace of that substance could be detected in those taken from other situations.

In anticipation of a system of defence, which was actually adopted, some earth taken from above, from beneath, and from each side of M———'s coffin, was analysed, and was found to be completely exempt from arsenic.—*Dublin Medical Press from Gazette Medicale.*

